# Metagenomic clustering reveals microbial contamination as an essential consideration in ultraconserved element design for phylogenomics with insect museum specimens

**DOI:** 10.1002/ece3.8625

**Published:** 2022-03-18

**Authors:** Alex R. Van Dam, Javier O. Covas Orizondo, Athena W. Lam, Duane D. McKenna, Matthew H. Van Dam

**Affiliations:** ^1^ Department of Biology University of Puerto Rico Mayagüez Mayagüez Puerto Rico; ^2^ 7143 Department of Entomology California Academy of Sciences San Francisco California USA; ^3^ Department of Biological Sciences University of Memphis Memphis Tennessee USA; ^4^ Center for Biodiversity Research University of Memphis Memphis Tennessee USA

**Keywords:** contamination, insects, museum specimens, ultraconserved elements

## Abstract

Phylogenomics via ultraconserved elements (UCEs) has led to improved phylogenetic reconstructions across the tree of life. However, inadvertently incorporating non‐targeted DNA into the UCE marker design will lead to misinformation being incorporated into subsequent analyses. To date, the effectiveness of basic metagenomic filtering strategies has not been assessed in arthropods. Designing markers from museum specimens requires careful consideration of methods due to the high levels of microbial contamination typically found in such specimens. We investigate if contaminant sequences are carried forward into a UCE marker set we developed from insect museum specimens using a standard bioinformatics pipeline. We find that the methods currently employed by most researchers do not exclude contamination from the final set of targets. Lastly, we highlight several paths forward for reducing contamination in UCE marker design.

## Graphical abstract

We investigate if genomic bacterial and laboratory contamination is introduced into ultraconserved element (UCE) probe design from five novel Coleoptera museum specimens. Our findings indicate that even very clean samples have contamination that is carried into the UCE probe design. These results indicate that metagenomic filtering should be a consideration in UCE probe design.

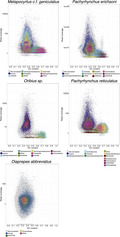

## 1 INTRODUCTION

Phylogenomic methods, including improvements to both sequencing and analytical techniques, have facilitated the resolution of long‐recalcitrant phylogenetic relationships across the tree of life (Blaimer et al., 2015; Brady et al., 2015; Brewer & Bond, 2013; Faircloth, 2016, 2017; Haddad et al., 2018; Lemmon et al., 2012; Locke et al., 2018; McCormack et al., 2012, 2016; McKenna et al., 2019; Misof et al., 2014; Van Dam et al., 2017). Ultraconserved elements (UCEs), conserved orthologous nuclear protein‐coding genes, and other phylogenomic markers have traditionally been developed from a wide variety of starting material, including fresh tissue for DNA or RNA marker development, ethanol‐preserved tissues, and museum specimens more than 100‐years‐old (Blaimer et al., 2016; Branstetter et al., 2017; Brewer & Bond, 2013; Derkarabetian et al., 2019; Locke et al., 2018; McGuire et al., 2018; Shin et al., 2018; Van Dam et al., 2017). Even formalin‐fixed specimens have shown promise for use in phylogenomic analyses (Hykin et al., 2015; Peacock et al., 2017; Ruane & Austin, 2017). It is generally assumed that using either newer tissues or published genomes, the amount of contamination from microbes and parasites will be so small as to be inconsequential and will therefore have little or no negative impact on phylogenomic marker development. Consequently, when freshly sourced tissue or genomes from NCBI are used, no significant metagenomic filtering is generally carried out (Faircloth, 2017; Gustafson et al., 2019).

However, such contaminants are common in older specimens, laboratory reagents, and the environment. Since phylogenetic analyses are likely to be affected by microbial contaminants, it is unsettling that this issue is not typically addressed in phylogenomic studies (Glassing et al., 2016; Hadfield & Eldridge, 2014; Sangiovanni et al., 2019). For larger taxa, it may be possible to simply use a single tissue type not known to host symbiotic microbes as the source of DNA (Łukasik et al., 2017; Seutin et al., 1991), but in smaller, soft‐bodied organisms, this may not be possible (e.g., Acari, micro‐Hymenoptera, Coccoidea, Nematoda, protists). Additionally, some taxa, including many insects, contain symbionts whose bacteriome may be nearly impossible to physically remove (McKenna, 2020).

Although filtering of metagenomic contaminants from UCE bait‐capture experiments has been performed (Bossert & Danforth, 2018), this has only been done after the target loci have been designed. Moreover, simply using a more stringent target cutoff will not remove baits that have inadvertently been designed from contaminants. Coverage‐based approaches work well with clean starting material, but when samples are old, coverage tends to be lower or uneven (Blaimer et al., 2016; McCormack et al., 2016; Van Dam et al., 2017). Other authors have developed pipelines to harvest UCE’s from exons (Van Dam et al., 2021), or simply blast scaffolds to exon accessions on NCBI. While both of these approaches are likely to reduce bacterial contamination, they will not reduce contamination from microbial eukaryotes in the tissue, and worse still, they rely on the assumption that loci containing exons retrieved by blast to the database of choice are not underlying mis‐annotations contributed by algorithm‐based gene annotation programs, as opposed to experimentally proven annotations. Presently, no standard metagenomic filtering strategy has been proposed as part of the UCE marker development workflow. This can be problematic when designing UCE markers from museum specimens or organisms whose tissues are routinely contaminated by symbionts or other organisms from the same environment.

Recent metagenomic filtering methods, such as canopy clustering and machine learning, rely on a reference database and multiple samples to create metagenomic bins (Alneberg et al., 2014; Eren et al., 2015; Nielsen et al., 2014; Nissen et al., 2018). While these methods are ideal, they are cost‐prohibitive for UCE marker pilot studies because they require many tens of specimens to deliver enough statistical power (Kang et al., 2015; Nielsen et al., 2014). Instead, three main strategies of metagenomic filtering are most commonly used with single specimen assemblies. These are (1) Metagenomic filtering of small specimens where symbionts are present. This method includes aligning reads to a reference genome of a model organism, for example, nematodes to *Caenorhabditis elegans*, or in ancient DNA studies on humans and many other animals, aligning reads back to the reference genome (Kumar et al., 2013; Rasmussen et al., 2015); (2) Examining nucleotide base composition bias, along with scaffold coverage, can also help cluster scaffolds (Kumar et al., 2013; Teeling et al., 2004); (3) Binning of scaffolds by taxonomic proximity and presence of exons in the hope that results provide the best guess as to taxonomic identity (Huson et al., 2018; Wood et al., 2019).

Binning scaffolds into taxonomic groups by genetic distance, base composition, and coverage should also have some merit for work with museum specimens; however, the degree of relatedness between non‐model organisms and their model anchor is a limiting factor. Even eukaryotic model organisms require metagenomic filtering to remove bacterial contamination (Fierst & Murdock, 2017). Throughout this paper, we refer to museum specimens as those specimens that are long preserved, poorly preserved, or dried and otherwise not from single tissue sources stored at or below −80°C. Because contamination may even exist in sequences from a reference genome (e.g., Koutsovoulos et al., 2016), the genomes derived from museum specimens of non‐model organisms will almost certainly require some metagenomic filtering. This is critical for phylogenomic marker development to prevent contamination from being represented in the probe set. To circumvent this problem, systematists, particularly those that study arthropods, typically try to anchor non‐model groups to a “clean” reference genome that is carefully extracted from fresh muscle tissue (Faircloth, 2017). However, “clean” material may not always be available in rare or small‐bodied arthropod groups. Thus, there is a need to establish best practices for developing phylogenomic markers from tissues that may be contaminated.

We explored the taxonomic composition of low‐coverage genome assemblies from insect museum specimens to produce a UCE probe set for the jewel weevils, tribe Pachyrhynchini (Coleoptera: Curculionidae: Entiminae). We then used these data to assess how metagenomic filtering affected UCE probe design. UCE alignments were constructed to determine whether anchoring UCEs to a base draft genome obtained from muscle tissue, as is standard practice, significantly reduces or eliminates contamination in the resulting probe design. If it does, the resulting UCEs should only contain endogenous insect DNA with few to no scaffolds originating from contaminant taxa. We describe our methods and findings below to investigate contamination levels resulting from using a clean draft genome to anchor museum specimens to develop a custom UCE marker set.

## 2 MATERIALS AND METHODS

### 2.1 Taxon sampling

Pachyrhynchini weevils are a charismatic group of beetles containing more than 155 species, with their center of diversity in the Philippines and Australasia (Rukmane, 2018). They are large and flightless, have aposematic coloration, and are ideal for investigating the evolution of island‐endemism and biogeography of the Philippines’ endemic fauna because of the large number of closely related species showing intriguing patterns of genetic and morphological divergence and (in contrast) convergent morphological evolution in allopatry and parapatry (Wang et al., 2018). While there are other phylogenomic markers for beetles (Faircloth, 2017; Haddad et al., 2018; Johnson et al., 2018; Shin et al., 2018), a phylogenomic marker set specifically tailored for optimal loci capture in the Pachyrhynchini does not yet exist. Here, we use three weevil species from the Philippines, one from Papua New Guinea (PNG), and a fourth species as an outgroup. The outgroup, *Diaprepes abbreviatus*, is an entimine weevil native to the Caribbean island of Puerto Rico. It is an economically important pest of a variety of fruit trees in the Caribbean region and the southern United States (Simpson et al., 1996). The addition of this outgroup taxon will ensure that the marker set is universal across the ingroup (Faircloth, 2017), and will likely also ensure utility across many other weevils in the extremely large subfamily Entiminae (more than 12,000 described species).

### 2.2 Collection localities

*Oribius* sp.: Papua New Guinea, Mt. Wilhelm, −5.714639 145.275 1680 m 7–8‐XI‐2014 leg. M Van Dam: *Pachyrhynchus* spp. and *Metapocyrtus* sp.: Makiling Forest Reserve Laguna, Calamba, Laguna, Philippines 2011. Pachyrhynchini samples were initially preserved in 95% EtOH upon collection in 2011, stored at room temperature, then extracted in 2017. The *Oribius* sp. Marshall, 1956 (placed in the tribe Celeuthetini) sample was preserved in 95% EtOH in 2014 and kept at ambient temperature while in the field for 5 weeks, and then preserved at −20°C until DNA extraction in 2017. *Diaprepes abbreviatus* (DDM2014024) was obtained by DDM from a laboratory research colony maintained by Dr. S. L. Lapointe in 2013. USDA/ARS, Horticultural Research Laboratory. 2001 South Rock Road. December 2013.

### 2.3 DNA extraction

For all DNA extractions only one sample was used to build downstream libraries and sequencing. Except for *D*. *abbreviatus*, DNA was extracted using DNeasy Tissue kits (Qiagen; Macherey‐Nagel) following the methods of Van Dam et al. (2017). Briefly, using sterilized forceps (soaked in 10% bleach and flame sterilized between use). Muscle tissue was removed from the pronotum, mesothorax, and abdomen and placed in a 1.5–2.0 ml centrifuge tube for tissue lysis and extraction (Van Dam et al., 2017). A single adult *D*. *abbreviatus* (DDM2938) was placed live in cold RNAlater and stored at −80 °C until extraction. Total genomic DNA was extracted from the prothorax and one hindleg using the G‐Biosciences Omniprep gDNA extraction kit/protocol and treated with RNAse A before genomic DNA library preparation following the methods of Shin et al. (2018).

### 2.4 Illumina library preparation and sequencing

#### 2.4.1 Diaprepes abbreviates

Following visualization on a 1× agarose gel, samples were sonicated to a size of approximately 400–700 bp on a Covaris M220 ultrasonicator (Covaris). Dual‐indexed shotgun genomic libraries were prepared by the University of Illinois Roy J. Carver Biotechnology Center/W.M. Keck Center (Champaign‐Urbana, Illinois) using the Hyper Library construction kit from Kapa Biosystems following manufacturer's instructions. All libraries were constructed using six cycles of PCR and size selected for fragments 400 to 500 bp in length. Paired‐end (PE) sequencing was conducted on a MiSeq v2 (250 bp PE reads) at the University of Illinois and using a HiSeq 2500 (150 bp PE reads) at Florida State University (Tallahassee, FL). Paired‐end SRA reads in FASTQ format were trimmed to remove low‐quality bases and adapter sequences and used as the input file for de novo assembly using the CLC Genomics Workbench version 4.9 (www.qiagenbioinformatics.com/products/clc‐genomics‐workbench/). Statistical parameters were maintained at their defaults.

#### 2.4.2 Other taxa

Following visualization on a 1× agarose gel, samples were sonicated to a size of approximately 400 bp on a Covaris M220 ultrasonicator (Covaris). Library preparation procedures followed Van Dam et al. (2017). Sequencing was performed via an Illumina HiSeq4000 at GeneWiz Next Generation Sequencing Center (South Plainfield, New Jersey).

#### 2.4.3 Illumina data quality filtering

Data were first inspected via a *fastqc* (Andrews, 2010) quality report. Data were then quality filtered with *Trimmomatic* v. 0.36 (Bolger et al., 2014) using the settings “PE: ILLUMINACLIP: TruSeq3‐PE.fa:2:30:10:2:keepBothReads LEADING:3 TRAILING:3 SLIDINGWINDOW:4:20 MINLEN:36.” A summary of the quality‐filtered reads, organized by sample, can be found in Table S1.

#### 2.4.4 Nanopore sequencing

Because the *Oribius* sp. sample was the most recently preserved, an attempt was made to add additional long reads to improve the assembly. This sample was not fragmented and went directly to library construction following the protocols in Oxford Nanopore Technologies (ONT) 1D PCR barcoding genomic DNA (SQK‐LSK108) for version R9 chemistry to construct the libraries. We followed the ONT protocol for the SpotOn Flow Cell version R9 chemistry (ONT cat No. FLO‐MIN 107 R9). The Library Loading Bead kit (ONT cat No. EXP‐LLB001) was used to help load samples onto the flow cell. The flow cell was loaded onto an ONT MinION sequencer and ran for 48 hours using ONT *MinKNOW* software.

#### 2.4.5 Nanopore data quality filtering

We used the *albacore* basecaller v2.1.3 (Oxford Nanopore Technologies, 2017a) to convert the fast5 data to FASTQ format (Oxford Nanopore Technologies, 2017b). Quality filtering was executed in *NanoFilt* 2.6.0 as part of *NanoPack* (De Coster et al., 2018). Read length, Phred quality scores, and other summary statistics were calculated using *Pauvre* (Schultz, 2018) and *NanoPlot*. A total of 171,816 and 87,639 reads met al*bacore* quality standards on two flow cells. These data were combined with the Illumina data (Table S1) to generate a hybrid assembly. The ONT reads were then used in the gap closer process with the *SPAdes* assembler (Bankevich et al., 2012).

#### 2.4.6 Draft genome assembly

*Kmergenie* (Chikhi & Medvedev, 2014) was used to find the optimal *k*‐mer size for assembly. The resulting optimal *k*‐mer size was then added to *k* values of 21,33,55 and 77 in the *SPAdes*‐*3*.*11*.*1* (Bankevich et al., 2012) genome assembly pipeline. *BUSCO* v1.22 (Seppey et al., 2019; Simão et al., 2015) was used to assess draft genome completeness of capturing conserved single‐copy genes using the Arthropoda Odb10 database. For summary statistics on assembly quality *bbmap stat*.*sh* (Bushnell, 2015) script was used to assess the quality of draft genome assemblies.

### 2.5 Metagenomic clustering strategy to explore sources of contamination

#### 2.5.1 Metagenomic filtering

Metagenomic filtering from a single library per sample necessitates using methods relying on scaffold coverage cutoff, GC content, and annotating the scaffolds via blast. The *Blobology* pipeline used here employs read coverage, *blast*+ (Kent, 2012) to the *nt* database for taxonomic annotation (*blast* v.2.7.1, *nt* database March 2019), and GC content to inform manual metagenomic clustering of assembly taxonomic annotation (Kumar et al., 2013). *Blast*+ settings were *blastn megablast* with an e‐value of 1e‐5 cutoff. Reads that passed the quality trimming step from Trimmomatic were aligned to their respective genome using the *mem* algorithm in *bwa* version *0*.*7*.*3a*, with default settings used to get an estimate of contig coverage and generate coverage plots (Li & Durbin, 2009). Plots produced via *Blobology* (Kumar et al., 2013) or *BlobTools* (Laetsch & Blaxter, 2017) assisted with annotation. This plotting method has proven successful in a wide variety of Eukaryotic genome sequencing projects for exploring potential sources of contamination and host‐symbiont relationships (Dentinger et al., 2016; Husnik & McCutcheon, 2016; Szitenberg et al., 2017). The blob graphs allow for the visualization of the relative degree of contamination in each draft genome, where scaffolds clustered based on taxonomy, coverage, and GC content (Figure 2).

#### 2.5.2 UCE marker development

We used the PHYLUCE v 1.5.0 pipeline (Faircloth, 2016) to develop UCEs from our five weevil genome assemblies. We used the *D*. *abbreviatus* draft genome as the base genome for this analysis. The *D*. *abbreviatus* genome was repeat masked using *RepeatMasker* 4.0.6 (Smit et al., 2015). A UCE combined probe length of 160 bp was used for probe development. UCE probes were culled to have an ≥80% match across the ingroup taxa. Duplicated probes mapped via *Lastz* (Harris, 2007) to multiple regions in their host genomes were removed (Faircloth, 2016). This resulted in 9217 UCEs and 86,079 master UCE probes. Probes were then mapped back to all five draft genomes via PHYLUCE and *Lastz* (Harris, 2007). The final data matrix was composed of genomic regions matching the master‐UCE probes with 80% coverage and 80% match across the five taxa with 500 bp flanking regions.

#### 2.5.3 UCE taxonomic annotation and metagenomic binning

The taxonomic mapping from the initial *blast*+ mapping was used to provide an initial putative taxonomic assignment to UCE‐based scaffolds (Figure 2). *Blatq* (Henderson, 2018; Kent, 2002, 2012) was used to rapidly align the UCEs to each genome (Figure 2). This initial taxonomic annotation revealed many scaffolds that remained unannotated.

#### 2.5.4 UCE probe parent scaffold annotation

Briefly, three methods were chosen as binning strategies for UCE parent scaffolds using this custom database (1) *blast*+ alignment‐based mapping (2) *Kraken2* k‐mer‐based annotation (3) *Lastz* nucleotide similarity‐based cutoff mapping. In more detail, the UCE loci's parent scaffolds were identified via the *Lastz* PHYLUCE results.

Using the three methods described above, scaffolds were assigned a taxonomic identity using a custom reference database. The custom references included the Rice Weevil genome v‐2.0 (*Sitophilus oryzae*, NCBI RefSeq:13876818) and the *Kraken2* (Wood et al., 2019) bacterial and fungal databases, as well as additional representative genomes from likely contaminant clades that were identified by the initial *blast*+ *nt* analysis. These clades were Acari, Nematoda, Platyhelminthes, Chordata, and Viridiplantae. A complete list of contaminating taxa used in the custom database is provided in Appendix S1: Table S4.

Using the custom reference set, the UCE‐based scaffolds were binned via *Kraken2* using default settings (Wood et al., 2019). Using the *Blast v 2*.*7*.*1* tool, “*blastn megablast”* was used to report only the annotation with the highest bit‐score E‐5 cut‐off. *Lastz* alignment tool was used with default settings and an 85% alignment cut‐off (as in Bossert & Danforth, 2018).

Finally, a linear regression model was used to see if *k*‐mer coverage was predicted by UCE parent scaffold length and contaminant taxa via *R* 3.3.3 (Cran, 2010). As short scaffolds are expected to contain more contaminants, the linear regression was used to help visualize contaminants by parent UCE scaffold size and coverage.

## 3 RESULTS

### 3.1 Illumina and nanopore sequencing and data quality filtering

After sequencing the Pachyrhynchini species, there were between 109 M and 122 M paired‐end reads (post *Trimmomatic* quality filtering) for each sample. The Nanopore sequencing produced 171,816 and 87,639 reads with a Q5 quality score or better on the two flow cells. See Appendix S1: Tables S1 and S2 and Figure S1 for a full summary of sequencing results.

### 3.2 Draft genome assembly

The Pachyrhynchini assemblies contained between 1.5 M and 0.5 M contigs, with an N50 ranging from 370–2189 bp, revealing that the assemblies are generally low quality (see Appendix S1: Table S3 for a full summary). *BUSCO* scores were also low ranging from 60 to 732 complete single‐copy genes out of 1367 single copy genes from the Arthropoda Odb10 database (see Appendix S1: Figure S2 for full *BUSCO* score summary). The relatively low quality is acceptable for building UCEs and is expected from museum specimens (Locke et al., 2018; Van Dam et al., 2017, 2019). The *D*. *abbreviatus* draft genome was highly fragmented, with 3.8 M contigs and an N50 of 437 (Appendix S1: Table S3), the BUSCO score of 164 was also low (Appendix S1: Figure S2).

### 3.3 Metagenomic clustering

**FIGURE 1:**
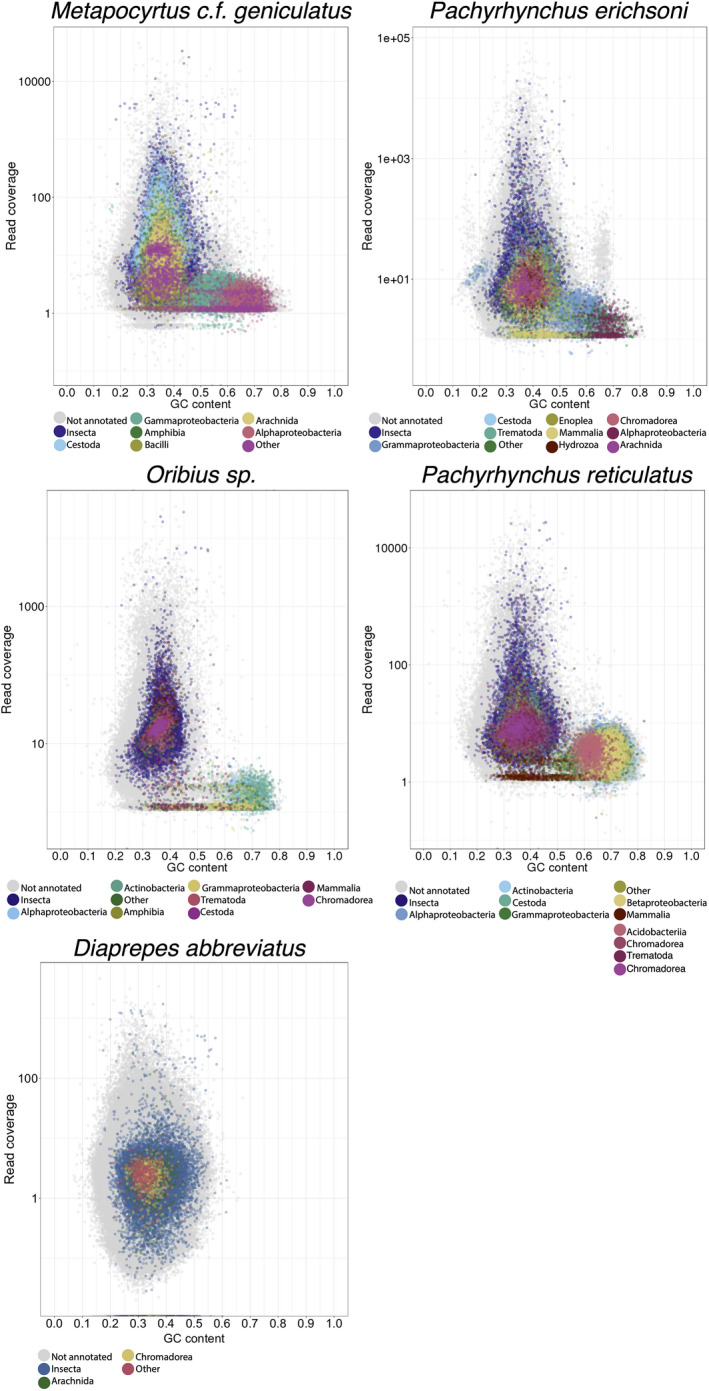
Blob plots of draft genome assembly scaffolds, *Y*‐axis is read coverage via the bwa‐mem algorithm, *X*‐axis is GC content of individual scaffolds. Color is coded by taxonomic class

The *Blobology* profile results revealed that every sample was heavily contaminated with bacterial contigs (Figure 1). There were two to three major clusters of Bacteria clades in each sample (Figure 1). The most common clades of Bacteria in the samples were as follows: Acidobacteria, Actinobacteria, α‐proteobacteria, β‐proteobacteria, and γ‐proteobacteria. Based on the *Blast*+ annotation in the Blob plots, it is clear that *Blast*+ retrieves a variety of spurious annotations (e.g., tropical amphibian lineages; see Figure 1). It is less clear whether some of the other eukaryotic annotations are spurious, such as Acari (mites) and Cestoda (parasitic flatworms) (Figure 1), because both mites and cestodes are common symbionts of beetles (Baumann, 2018; Shostak, 2014). Some scaffolds were annotated to Mammalia and could be spurious annotations and/or laboratory contamination (Figure 1). The *D*. *abbreviates* draft genome was quite clean compared to the other four taxa with very few contigs assigned to Bacteria, and no Bacteria contigs forming noticeable blob‐like clusters (Figure 1).

The bacterial contaminants are unlikely to be spurious since these formed distinct blobs that tended to have higher GC content than the insect contigs and less than 100x coverage. There is also a scattering of both insect and contaminant reads with high coverage and lower GC content (Figure 1). Also, there is a significant overlap of low coverage contigs between the eukaryote/insect blob and the bacteria blobs (Figure 1).

### 3.4 UCE markers and metagenomic UCE loci binning

**FIGURE 2:**
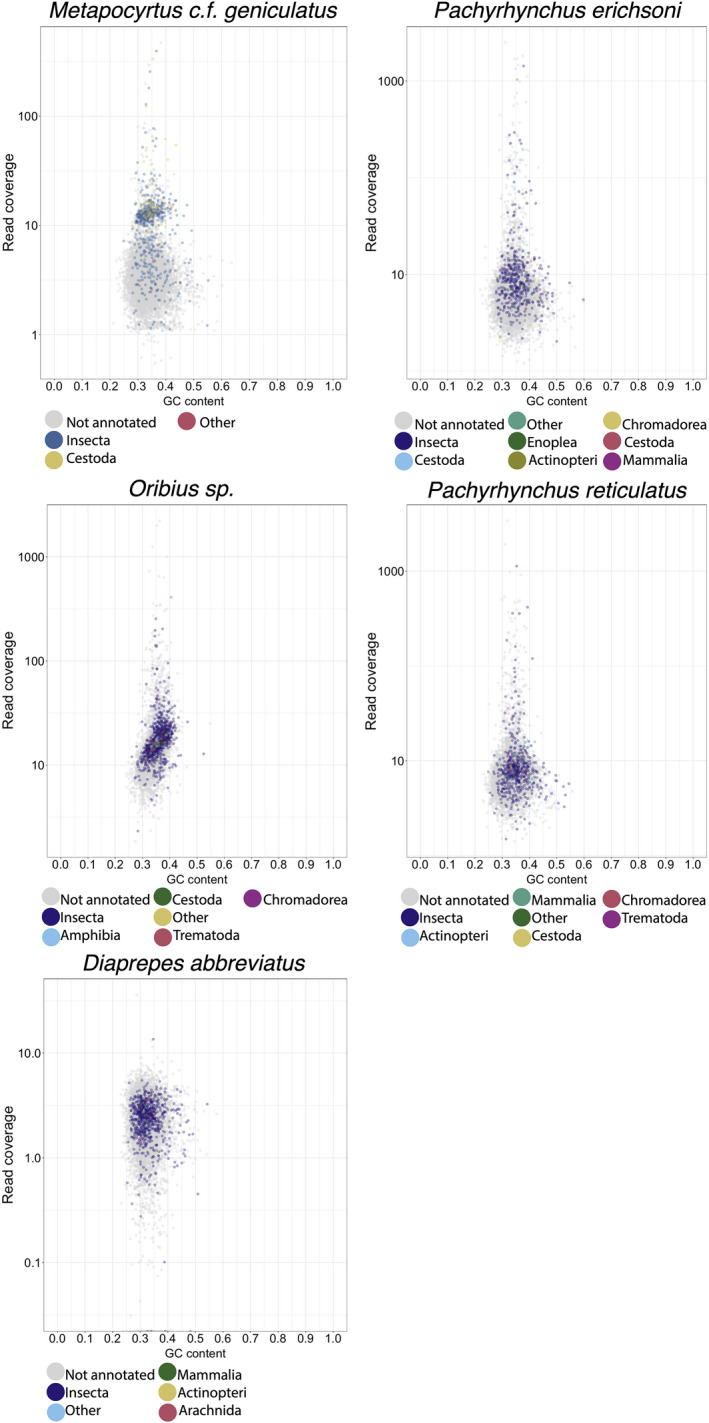
Blob plots of UCE‐bearing scaffolds from the master UCE probe set. The Y‐axis is read coverage via the bwa‐mem algorithm, and the *X*‐axis is the GC content of individual scaffolds. Color is coded by taxonomic class

**FIGURE 3:**
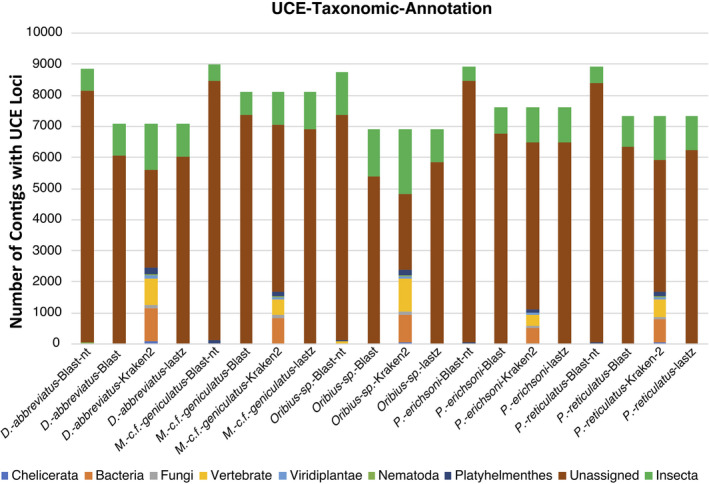
UCE bar chart. *Y*‐axis is the total number of UCEs, and the *X*‐axis is the method used to annotate associated species. Color is coded by taxonomic annotation

PHYLUCE produced 9217 UCE loci that were conserved across the five taxa. From this master‐probe list, there were 8688 UCE loci in the final matrix. The count of UCE loci that is comprised of the final matrix by taxon are as follows: 8404 *Metapocyrtus (Artapocyrtus) c*.*f*. *geniculatus*, 8073 *Pachyrhynchus erichsoni*, 7970 *Pachyrhynchus reticulatus*, 7780 *Oribius* sp., and 7732 *Diaprepes abbreviatus*. The rapid UCE scaffold annotation via *blatq* revealed that the final UCE dataset consistently mapped to various contaminating sequences, including Bacteria, Acari, and Cestoda, across all five weevil taxa (Figures 2 and 3).

**FIGURE 4:**
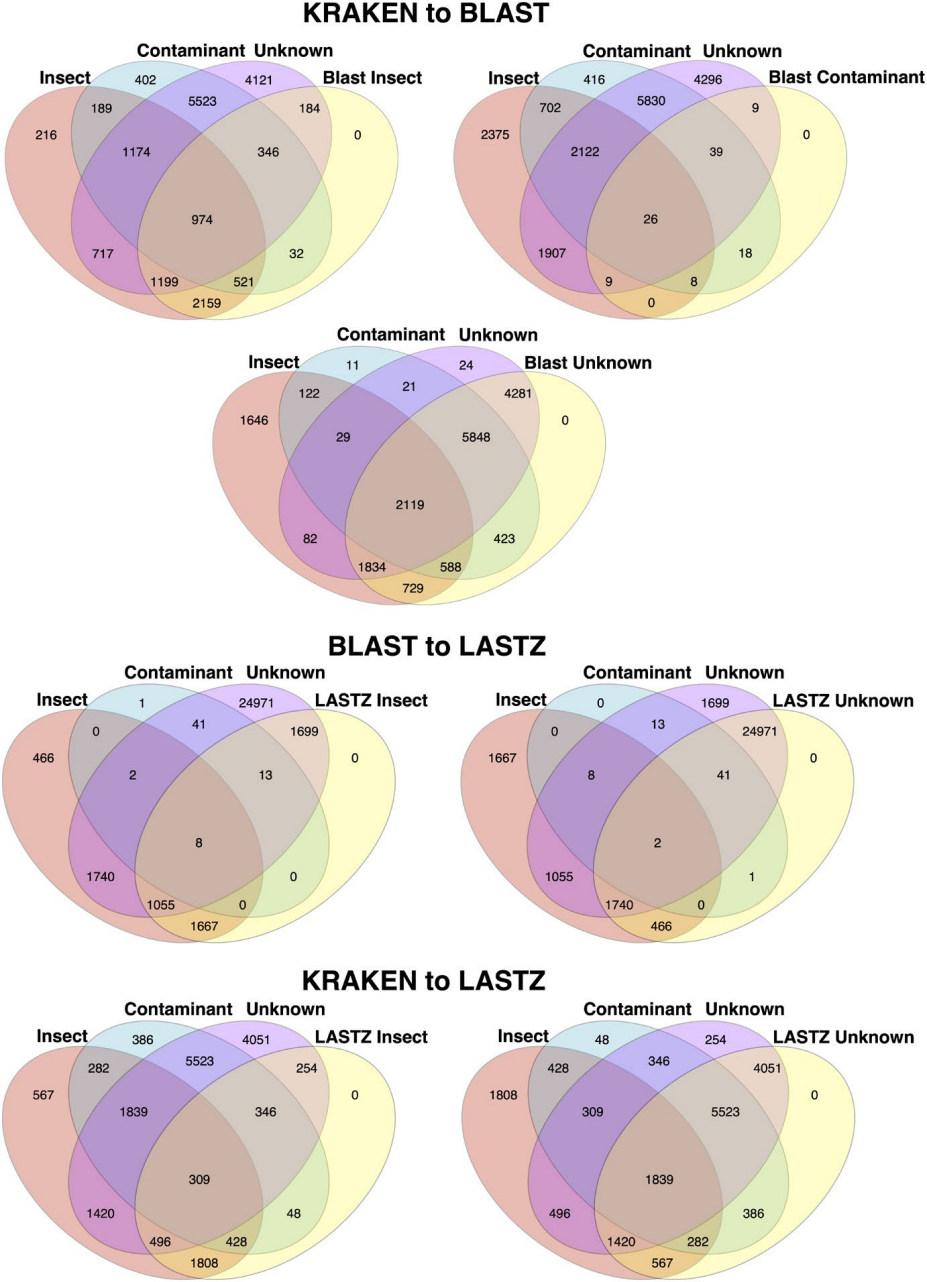
Venn diagrams of UCE bearing scaffolds annotated by taxonomic groups. The groupings are the same ones used in the final data matrix

The metagenomic binning via *Kraken2* of the UCE loci in the final data matrix also had significant bacterial contamination (Figure 3). The *Kraken2* annotation retrieved >160 UCE loci of Insecta for each of the five species compared to the *Blast*+ annotation to the same database (Figure 3). The Kraken2 binning revealed that all taxa had significant bacterial contamination (4%–10%; range 341–966 Bacteria UCE contigs Figure 3). The *blast*+ annotation with both the *nt* and custom database revealed that the UCE parent scaffolds had bacterial and/or fungal contamination across all five weevil species (see Figure 3). Similar results were obtained via the Kraken2 annotation but with more scaffolds identified as contaminants than *Blast*+ (Figure 3). While the lastz methods produced a consistent set of UCE scaffolds across the species, many were dubious because they overlapped with contaminant scaffolds using blast+and Kraken2 (Figures 3 and 4).

**FIGURE 5:**
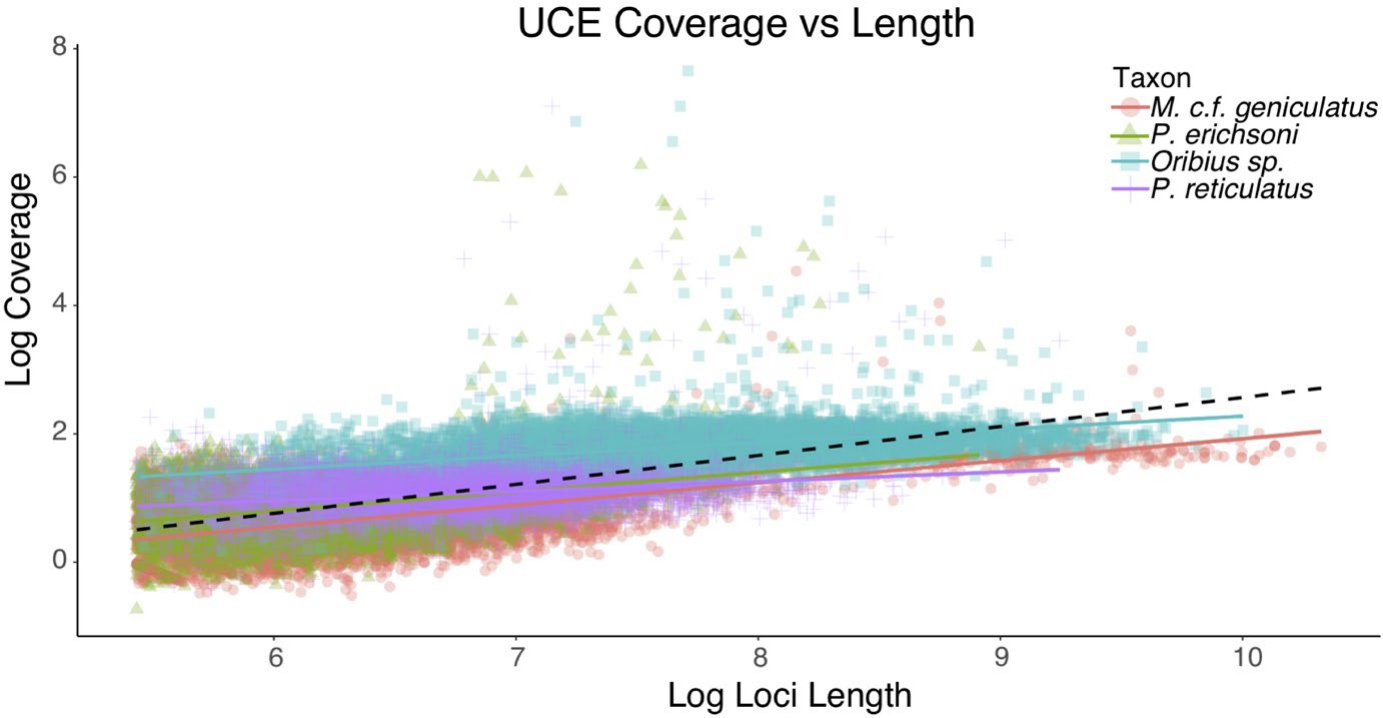
Linear regression of log UCE loci length versus the log of coverage for Pachyrhynchini taxa in the final data matrix

The linear regression model of UCE parent scaffolds length and k‐mer coverage did not show any significant breaks in coverage demarcating potential contaminants (Figure 5). Scaffolds of both contaminant UCEs and Insecta UCEs had lengths over 10 kb and over 100× coverage (Figure 5), making it impossible to discriminate between insect and non‐insect scaffolds by coverage/length alone. *K*‐mer coverage and scaffold length were also significantly correlated (*p*‐value: <2.2e−16).

## 4 DISCUSSION

In all five taxa, <50% of the UCE parent scaffolds had their origins definitively binned by either *blast*+, *lastz*, or *Kraken2* (Figure 2). We have demonstrated that despite careful extraction to avoid contamination, museum specimens of weevils consistently contain high levels of bacterial and non‐target contamination. This contamination was carried over to the UCE alignment matrix in the form of UCEs that reside in scaffolds of bacterial origin. The level of contamination varies by reference database (nt vs. custom) and the method of metagenomic binning (*blast*+, *Kraken2*, *lastz*). Contamination is consistent across all methods and databases via cross‐checking the methods against each other. The UCE contamination does not appear to be restricted to low coverage (<10×), short scaffolds (~1 kb), but appears to populate long scaffolds (>10 kb) with high coverage (>50×). In fact, many of the contaminant scaffolds that were incorporated into the UCE data matrix had high coverage and were relatively long. An artifact of de Bruijn graph assembly programs is that smaller genomes tend to have higher coverage (Kumar et al., 2013). While coverage may be an excellent method to eliminate low‐level laboratory contamination (Douglass et al., 2019), it is doubtful that contamination in museum specimens would be eliminated by coverage and length alone because of near‐complete coverage‐depth overlap in these specimens (Figures 1, 2 and 5). Simply running *blast*+ to an exemplar bacterial genome (e.g., *Wolbachia* or *E*. *coli*) or an exemplar eukaryote model organism (e.g., *Sitophilus orizae* or *Drosophila*) will produce hundreds to thousands of ambiguous un‐annotated scaffolds. Most of the systematics community works on organisms distantly related to model organisms, suggesting that this metagenomic binning approach will continue to be problematic across taxa. Because of the costly nature of phylogenomic studies, a reduction in loci is not ideal. Most studies that benchmark the accuracy and recall of metagenomic mapping/binning methods fail to consider the genetic distance between non‐model organisms and the data in the NCBI *nt* and RefSeq databases (Sarmashghi et al., 2019). This problem is manifested here, as the majority of contigs remained unannotated.

### 4.1 Paths forward for museum‐based insect phylogenomics

With the advent of truly high‐throughput Illumina sequencers (e.g., NovaSeq), the cost of producing draft genomes is greatly reduced, so it is possible to create relatively decent draft genomes and extract loci from a single museum specimen (Cotoras et al., 2017; Derkarabetian et al., 2019; McGuire et al., 2018; McKenna et al., 2019). The lower cost of sequencing will also make it possible to use more statistically robust methods, such as canopy clustering‐based methods, for example, *CONCOCT* or *MetaBAT*, assuming you have a close reference genome (Alneberg et al., 2014; Kang et al., 2015). Machine deep learning metagenomic binning methods (e.g., *VAMB*) should also be explored and could be a way to rescue these types of heterogeneous multi‐taxa data (Nissen et al., 2018). These methods could potentially work for studies entirely composed of museum specimens but remain untested. Another potential path is reference assemblies that incorporate Hi‐C data (Dudchenko et al., 2017). These data types group scaffolds into ordered chromosomes based on their Hi‐C mapping interactions. Consequently, genomes that do not map to the other chromosomes, such as bacteria, can easily be identified and removed.

Another possible way to reduce the chances of contamination is using Anchored Hybrid Enrichment (AHE) probe sets or other exon‐based markers (Bi et al., 2012; Haddad et al., 2018; Lemmon et al., 2012; Shin et al., 2018). With these methods, the initial probe set is anchored to single‐copy nuclear genes, ideally derived from transcriptomes. Anchoring marker sets to poly‐A tailed RNA‐seq data would certainly eliminate most if not all bacterial contamination but may still require screening for microbial eukaryotes. However, these methods typically require an annotated genome and/or transcriptomes for their design (Bi et al., 2012; Lemmon et al., 2012), limiting their use to clades where freshly collected material is available (i.e., not preserved museum specimens). To capture a diverse set of genomic categories, using UCEs for their intergenic and intronic markers (Van Dam et al., 2021) coupled with AHE seems to be a beneficial combination (Wood et al., 2018).

Several other aspects of contamination on UCE datasets remain unresolved. DNA degradation in museum specimens may further confound the accuracy of binning because longer scaffolds may be more accurately binned than shorter scaffolds (Leidenfrost et al., 2020). Examining the effects of UCE parent scaffold or contig length on the accuracy of metagenomic binning should also be explored in the future to potentially improve the accuracy of novel UCE marker set development. While we demonstrate that contaminants are incorporated into a UCE dataset, the effects of contamination on the subsequent phylogeny remain unexplored.

The potential contamination of UCE data used in phylogenomic papers involving museum specimens is not limited to insects. Similar levels of contamination are likely prevalent in other invertebrates and in herbaria or archeological specimens. The methods used by the systematics community have advanced rapidly in the era of phylogenomics. However, relatively little attention has been given to potential contamination in these datasets. Future studies should incorporate a robust metagenomic binning method (beyond just a blast+filtering step) to eliminate sources of contamination from downstream phylogenomic analyses.

## CONFLICT OF INTEREST

The authors have no conflicts of interest to declare.

## AUTHOR CONTRIBUTIONS

**Alex R. Van Dam:** Conceptualization (lead); Data curation (equal); Formal analysis (equal); Funding acquisition (equal); Investigation (equal); Methodology (equal); Project administration (equal); Resources (equal); Software (equal); Supervision (equal); Validation (equal); Visualization (equal); Writing – original draft (equal); Writing – review & editing (equal). **Javier O. Covas Orizondo:** Data curation (equal); Formal analysis (equal); Investigation (equal). **Athena W. Lam:** Data curation (equal); Investigation (equal); Methodology (equal); Project administration (equal); Resources (equal); Validation (equal); Writing – original draft (equal); Writing – review & editing (equal). **Duane D. McKenna:** Data curation (equal); Formal analysis (equal); Funding acquisition (equal); Resources (equal); Writing – original draft (equal); Writing – review & editing (equal). **Matthew H. Van Dam:** Conceptualization (equal); Data curation (equal); Formal analysis (equal); Funding acquisition (equal); Investigation (equal); Methodology (equal); Project administration (equal); Resources (equal); Software (equal); Supervision (equal); Validation (equal); Visualization (equal); Writing – original draft (equal); Writing – review & editing (equal).

## Supporting information

Appendix S1

## Data Availability

Raw data associated with this work can be accessed via NCBI BioProject ID PRJNA800670.
