# Supplementary material for: Metagenomic clustering reveals microbial contamination as an essential consideration in ultraconserved element design for phylogenomics with insect museum specimens
Source: Ecol Evol. 2022 Mar 18;12(3):e8625. doi: 10.1002/ece3.8625 (PMC8932080; doi:10.1002/ece3.8625)
Supplement: Supplementary file 1 — Appendix S1 [file ECE3-12-e8625-s001.pdf]

Supplementary Table 1.
Summary statistics of reads used in draft assemblies post Trimmomatic quality filtering and accepted by the internal default filtering standards of *SPAdes-3.11.1* of the Illumina reads used in draft assemblies.

Sample	PE Unfiltered Reads/Filtered PE Reads	Total bp Filtered Reads	Mean length Filtered Reads	95 CI length Filtered Reads	Minimum read length Filtered Reads	Maximum read length Filtered Reads	Median Filtered Reads
D. abreviatus	7905910 /7902470	1975617500	250	250	250	250	250
M. artapocyrtus	129642768 /125782895	17834735726	141.79	0.00	40	151	151
P. erichsnoni	115800304 /113778588	14101411037	123.94	0.00	40	151	130
P. reticulatus	113248026 /110922492	14389967103	129.73	0.00	40	151	150
Oribius sp. Illumina reads	125314394 /121575413	16157124237	132.09	0.00	40	151	150
Oribius sp. ONT and Illumina reads	125486210/ 121663052	16273427960	133.76	0.00	40	20083	151

Supplementary Table 2.
Summary statistics from Oxford Nanopore sequencing results from with quality filtered reads using *Oribius sp*. draft genome assembly.

	Unfiltered	NanoFilt Q10 Filtered
Mean read length:	1,322.10	1,327.1
Mean read quality:	9.9	10.8
Median read length:	934	946.0
Median read quality	10	10.8
Number of reads:	171,816.00	87,639
Read length N50:	1,641.00	1,637.00
Total bases:	227,163,436	116,303,723
	Unfiltered Number, percentage and megabases of reads above quality cutoffs	NanoFilt Q10 Filtered Number, percentage and megabases of reads above quality cutoffs
>Q5	171816 (100.0%) 227.2Mb	87639 (100.0%) 116.3Mb
>Q7	171767 (100.0%) 227.1Mb	87639 (100.0%) 116.3Mb
>Q10	87639 (51.0%) 116.3Mb	87639 (100.0%) 116.3Mb
>Q12	2385 (1.4%) 2.6Mb	2385 (2.7%) 2.6Mb

>Q15	0 (0.0%) 0.0Mb	0 (0.0%) 0.0Mb

Unfiltered Nanopore reads

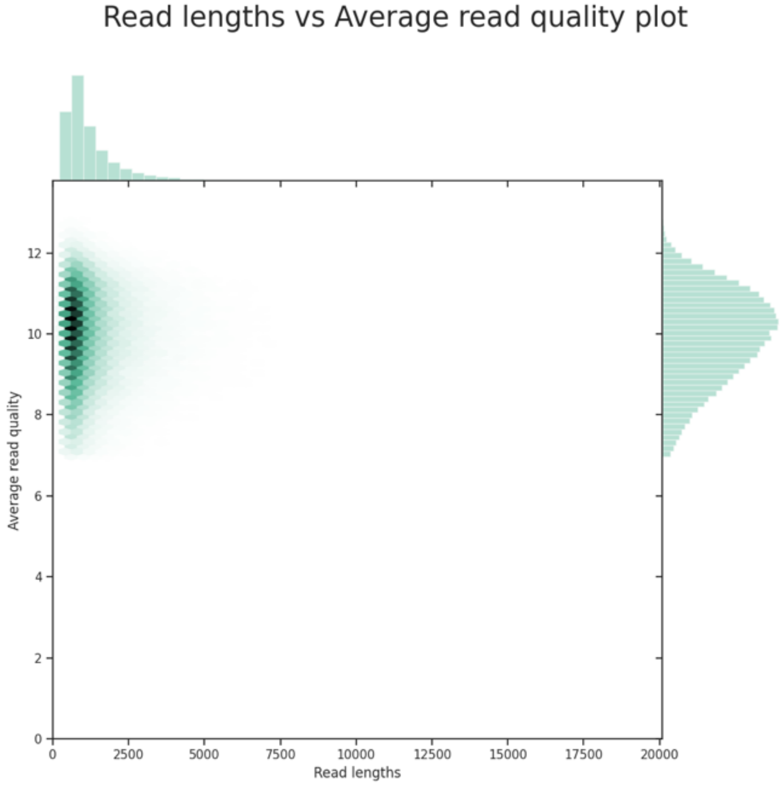

NanoFilt >Q10 Nanopore reads

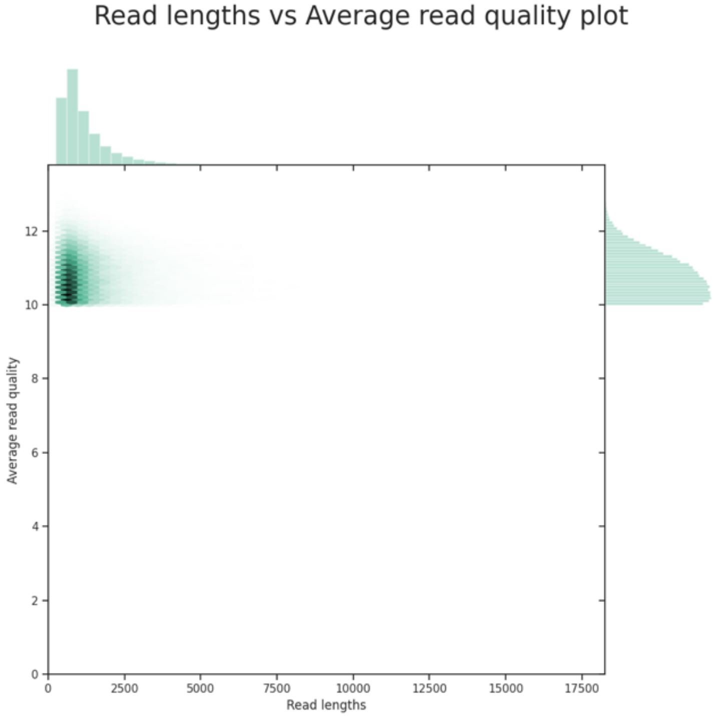

Supplementary Figure 1. *Oribius sp*. Oxford Nanopore sequencing results.

Supplementary Table 3.
Summary statistics of from BBMAP stats.sh script of draft genomes.

Species	*D. abbreviatus*	*M. artapocyrtus*	*P. erichsoni*	*Oribius sp.*	*P. reticulatus*
Main genome scaffold total:	3862772	1610785	1548369	529906	1734547
Main genome contig total:	3862772	1612124	1548394	530110	1734691
Main genome scaffold sequence total:	1202.063 MB	1156.021 MB	567.173 MB	667.428 MB	840.673 MB
Main genome contig sequence total:	1202.063 MB	1156.003 MB	567.172 MB	667.421 MB	840.670 MB
Main genome scaffold N/L50:	613470/437	172377/1.397 KB	499752/370	90998/2.19 KB	415710/553
Main genome contig N/L50:	613470/437	172734/1.395 KB	499765/370	91079/2.189 KB	416747/552
Main genome scaffold N/L90:	2815901/133	1114158/275	1274971/245	302873/612	1364794/259
Main genome contig N/L90:	2815901/133	1115041/275	1274989/245	303005/612	1364879/259
Max scaffold length:	16.718 KB	534.766 KB	26.632 KB	24.053 KB	24.755 KB
Max contig length:	16.718 KB	534.766 KB	26.632 KB	24.053 KB	24.755 KB
Number of scaffolds > 50 KB:	0	44	0	0	0
% main genome in scaffolds > 50 KB:	0.00%	0.40%	0.00%	0.00%	0.00%

Supplementary Table 4.
List of genome accession id's from RefSeq and WormBase added into the final database in addition to the Rice Weevil Genome, Kraken2 Fungal, and Kraken2 Bacterial databases.

caenorhabditis_elegans.PRJNA13758.WBPS14.genomic_masked.fa.gz.f
echinostoma_caproni.PRJEB1207.WBPS14.genomic_masked.fa.gz.fna
GCF_000313855.1_ASM31385v1_genomic.fna.gz.fna
GCF_002532875.1_vjacob_1.0_genomic.fna.gz.fna
gongylonema_pulchrum.PRJEB505.WBPS14.genomic_masked.fa.gz.fna
hymenolepis_diminuta.PRJEB507.WBPS14.genomic_masked.fa.gz
protopolystoma_xenopodis.PRJEB1201.WBPS14.genomic_masked.fa.gz.
schistosoma_curassoni.PRJEB519.WBPS14.genomic_masked.fa.gz.fna
schistosoma_mansoni.PRJEA36577.WBPS14.genomic_masked.fa.gz.fna
soboliphyme_baturini.PRJEB516.WBPS14.genomic_masked.fa.gz.fna

GCF_000001405.39_GRCh38.p13_genomic.fna

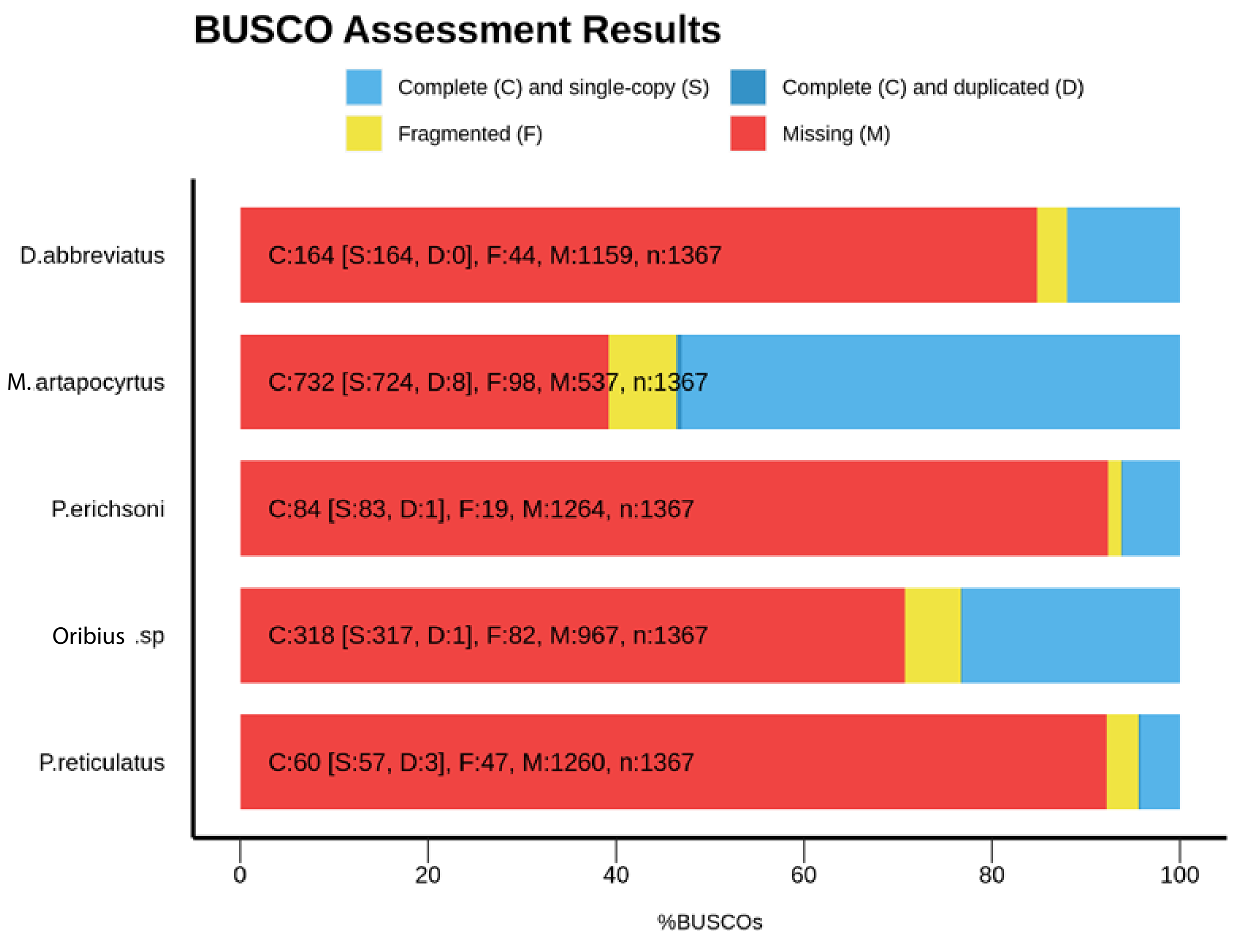

Supplementary Figure 2. BUSCO 3.0.2 results using the ArthropodaOdb10 database and fly as the Augustus species gene prediction model.
